# The Impact of Infection in Pregnancy on Placental Vascular Development and Adverse Birth Outcomes

**DOI:** 10.3389/fmicb.2019.01924

**Published:** 2019-08-22

**Authors:** Andrea M. Weckman, Michelle Ngai, Julie Wright, Chloe R. McDonald, Kevin C. Kain

**Affiliations:** ^1^Department of Laboratory Medicine and Pathobiology, University of Toronto, Toronto, ON, Canada; ^2^SAR Laboratories, Sandra Rotman Centre for Global Health, University Health Network-Toronto General Hospital, Toronto, ON, Canada; ^3^Tropical Disease Unit, Division of Infectious Diseases, Department of Medicine, University of Toronto, Toronto, ON, Canada

**Keywords:** infection, pregnancy, placenta, vascular development, adverse birth outcomes

## Abstract

Healthy fetal development is dependent on nutrient and oxygen transfer *via* the placenta. Optimal growth and function of placental vasculature is therefore essential to support *in utero* development. Vasculogenesis, the *de novo* formation of blood vessels, and angiogenesis, the branching and remodeling of existing vasculature, mediate the development and maturation of placental villi, which form the materno-fetal interface. Several lines of evidence indicate that systemic maternal infection and consequent inflammation can disrupt placental vasculogenesis and angiogenesis. The resulting alterations in placental hemodynamics impact fetal growth and contribute to poor birth outcomes including preterm delivery, small-for-gestational age (SGA), stillbirth, and low birth weight (LBW). Furthermore, pathways involved in maternal immune activation and placental vascularization parallel those involved in normal fetal development, notably neurovascular development. Therefore, immune-mediated disruption of angiogenic pathways at the materno-fetal interface may also have long-term neurological consequences for offspring. Here, we review current literature evaluating the influence of maternal infection and immune activation at the materno-fetal interface and the subsequent impact on placental vascular function and birth outcome. Immunomodulatory pathways, including chemokines and cytokines released in response to maternal infection, interact closely with the principal pathways regulating placental vascular development, including the angiopoietin-Tie-2, vascular endothelial growth factor (VEGF), and placental growth factor (PlGF) pathways. A detailed mechanistic understanding of how maternal infections impact placental and fetal development is critical to the design of effective interventions to promote placental growth and function and thereby reduce adverse birth outcomes.

## Introduction

Each year an estimated 20 million infants are born low birth weight (LBW) (<2,500 g) and 14.9 million are born preterm (Lee et al., 2013). Preterm birth (PTB) is the leading direct cause of under 5 mortality, responsible for more than 1 million deaths per year (Liu et al., 2016). According to the Global Burden of Disease Study, the disability-adjusted life years attributable to PTB is 77 million, comparable to the estimates for HIV or malaria (Murray et al., 2012). While these adverse birth outcomes predominantly occur in low- and middle-income countries (Blencowe et al., 2012), rates are increasing globally, and have been consistently linked with increased risks of long-term health consequences for offspring including cardiovascular disease, diabetes, obesity, and neurodevelopmental disorders (Bilbo and Schwarz, 2009; Calkins and Devaskar, 2011). The burden of infectious diseases in pregnancy (e.g., malaria, HIV, sexually transmitted infections) is also highest in low- and middle-income countries, and a growing body of evidence indicates that these prevalent infections contribute to poor birth outcomes by inflammation-mediated disruption of placental development and function (Watson-Jones et al., 2002; Chico et al., 2017; Conroy et al., 2017; McDonald et al., 2018, 2019).

Fetal development is governed by tightly regulated processes at the materno-fetal interface. Placental vasculogenesis and angiogenesis mediate placental vascular development, which is critical to nutrient and oxygen delivery to the developing fetus. These processes are primarily regulated by mediators in the vascular endothelial growth factor (VEGF) and angiopoietin families (Geva et al., 2002). Dysregulation of these factors is associated with inadequate placental vascularization, leading to hemodynamic placental insufficiency, inadequate delivery of nutrients and oxygen to the fetus, and consequently adverse birth outcomes (Kaufmann et al., 2003). Inflammatory and angiogenic pathways are interdependent and co-regulatory, suggesting that the host response to maternal infection could dysregulate pathways essential for placental vascular development. Here, we review the impact of systemic maternal infections resulting in immune activation at the materno-fetal interface – and its subsequent impact on placental vascularization – adverse birth outcomes, and later-life neurocognitive deficits in offspring.

## Placental Development: Vasculogenesis and Angiogenesis

The placenta forms the primary interface between mother and fetus, and a healthy functioning placenta is essential for a successful pregnancy. The placenta is a multi-function organ, acting as the site of materno-fetal nutrient, oxygen and waste exchange; producing hormones and growth factors critical for pregnancy progression and maintenance; and acting as a barrier to protect the fetus from maternal immune attack, toxins, and infectious pathogens (Wang and Zhao, 2010). These functions all rely on proper vascularization and perfusion of the placenta, and disruptions to placental vascular development and adaptation are associated with adverse pregnancy outcomes including preeclampsia, small-for-gestational age (SGA), PTB, and stillbirth (Kingdom, 1998; Gagnon, 2003; Wang and Zhao, 2010; Romero et al., 2011; Conroy et al., 2013; Morgan, 2016; Silver, 2018).

Placental vascular development begins early in pregnancy and undergoes adaptations across gestation. On the maternal side, uteroplacental circulation is established by the end of the first trimester (Wang and Zhao, 2010). Maternal vascular adaptation involves remodeling of the uterine spiral arteries by invasive fetal-derived extravillous trophoblasts to enable low-resistance blood flow into the intervillous space of the placenta (Wang and Zhao, 2010; Pollheimer et al., 2018). On the fetal side, primary placental villi begin to develop around day 13 post-conception, and fetoplacental vascularization of villi begins around 21 days post-conception (Kingdom et al., 2000; Kaufmann et al., 2004; Demir et al., 2006). The tertiary villi around which maternal blood flows in the intervillous space act as the functional units of the materno-fetal interface. Fetal-derived syncytiotrophoblasts are the primary mediators of exchange, protein-production, and defense at the materno-fetal interface.

Vascularization of placental villi involves the sequential processes of vasculogenesis and angiogenesis. Vasculogenesis is the *de novo* formation of blood vessels *via* differentiation of mesenchymal cells to hemangiogenic stem cells and then endothelial precursors (Demir et al., 2007). The VEGF family of ligands and receptors are heavily involved in the regulation of both vasculogenesis and angiogenesis. VEGF and its receptors (VEGFR-1 and -2) are expressed very early in placental development, and the production of VEGF by cytotrophoblasts and Hofbauer cells is thought to drive early placental vasculogenesis and subsequent angiogenesis (Kaufmann et al., 2004; Demir et al., 2006, 2007).

**Figure 1:**
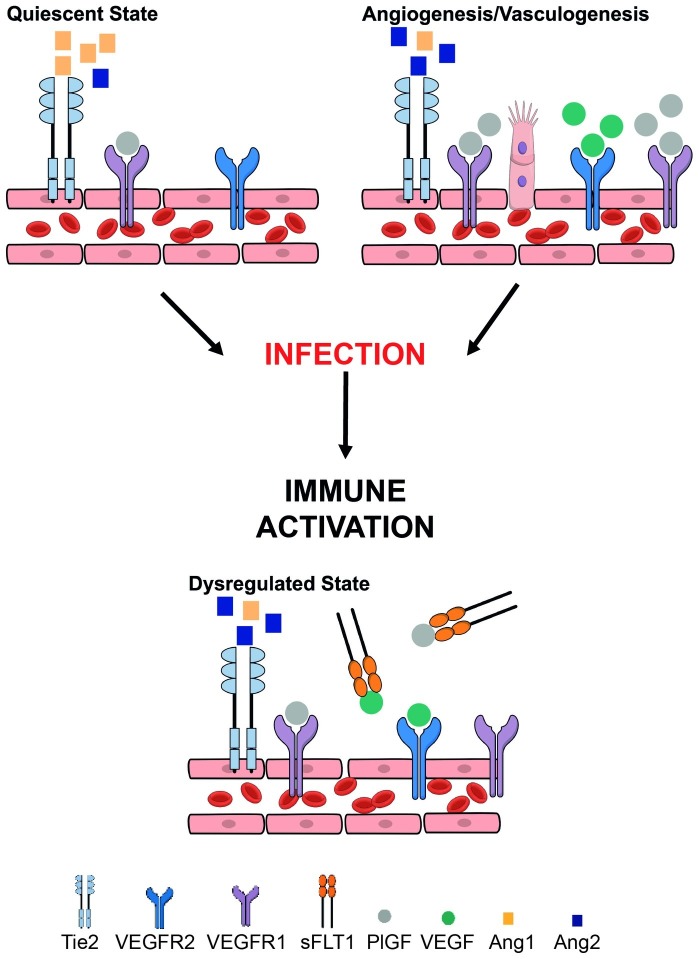
Overview of key angiogenic and vasculogenic factors mediating placental function and how they may be disrupted in the context of maternal infection. Placental vasculogenesis and angiogenesis are processes that are vital for placental vascular development and function. These processes depend on a fine balance between pro-angiogenic and anti-angiogenic pathways. The vascular endothelial growth factor (VEGF) family of proteins (including PlGF – placental growth factor) are pro-angiogenic mediators. VEGF and PlGF bind VEGF receptor 1 [fms-like tyrosine kinase (Flt-1)] to induce vessel proliferation and sprouting. Alternative splicing of Flt-1 results in soluble Flt-1 (sFlt-1) that is anti-angiogenic. Angiopoietin-1 (Ang-1) binds its tyrosine-kinase receptor Tie2 inducing vessel maturation, whereas angiopoietin-2 (Ang-2) promotes vessel destabilization and angiogenesis. Tight control of these pathways is essential for proper vascular development, remodeling, robust placental function, and healthy birth outcomes. Maternal infection (e.g., malaria, HIV-1) can result in immune activation and inflammation which dysregulates these tightly regulated processes, contributing to poor birth outcomes.

Angiogenesis begins at approximately 32 days post-conception (Kaufmann et al., 2004). From this point until term, the placental vascular network needed to support the rapidly growing fetus is built predominantly *via* branching and non-branching angiogenesis. The molecular mediation of angiogenesis requires tight temporal and spatial coordination and interaction between VEGF and angiopoietin protein family signaling (Ahmed and Perkins, 2000; Carmeliet, 2000; Yancopoulos et al., 2000; Geva et al., 2002; Charnock-Jones et al., 2004; Kaufmann et al., 2004; Benirschke et al., 2012). VEGF, placental growth factor (PlGF), and their inhibitor soluble fms-like tyrosine kinase-1 (sFlt-1) are produced by trophoblasts, and their balance is critical to healthy placental vascular development (Ahmed and Perkins, 2000; Kingdom et al., 2000; Charnock-Jones et al., 2004; Kaufmann et al., 2004). The timing and ratio of angiopoietin-1 (Ang-1) and its antagonist angiopoietin-2 (Ang-2) signaling through their receptor Tie2 is also essential for placental vascularization. Ang-1 promotes vascular maturation and stability, whereas Ang-2 allows for the destabilization and endothelial plasticity required for VEGF to drive angiogenesis and vascular remodeling (Carmeliet, 2000; Yancopoulos et al., 2000; Geva et al., 2002). These functions are reflected in the longitudinal dynamics of Ang-1 and -2 in healthy pregnancies: Ang-1 is initially low and increases across pregnancy as placental vasculature becomes more established, while Ang-2 decreases across pregnancy (Geva et al., 2002). Several groups have hypothesized that the tightly regulated longitudinal dynamics of VEGF and PlGF, as well as Ang-1 and -2, provide a molecular basis for the temporal transition from vasculogenesis to branching and then non-branching angiogenesis that underlies placental vascular development (Figure 1; Ahmed and Perkins, 2000; Geva et al., 2002; Kaufmann et al., 2004).

VEGF, PlGF, and VEGFR-1 are expressed on extravillous and villous trophoblasts, as well as Hofbauer cells in human placentas (Charnock-Jones et al., 1994; Ahmed et al., 1995; Clark et al., 1996; Khaliq et al., 1996; Vuorela et al., 1997). Their signaling has a role in trophoblast function including proliferation, differentiation, and nitric oxide (NO) production (Charnock-Jones et al., 1994; Ahmed et al., 1997; Athanassiades et al., 1998; Athanassiades and Lala, 1998; Khaliq et al., 1999). Ang-1 and -2 and their receptor Tie2 are also expressed in villous and extravillous trophoblasts in specific cell-type and temporal patterns across pregnancy, and *in vitro* studies reported a role for Ang/Tie2 signaling in trophoblast NO production and migration (Dunk et al., 2000; Goldman-Wohl et al., 2000; Seval et al., 2008).

Collectively, these data support critical roles for the VEGF and angiopoietin pathways in both fetoplacental vascularization (i.e., vasculogenesis and angiogenesis in the villi) and trophoblast function, as well as uteroplacental remodeling (i.e. trophoblast-mediated maternal spiral artery remodeling). With such diverse and interdependent roles for angiogenic factors across placental development, it is not surprising that their dysregulation has been associated with pathologic pregnancies and adverse birth outcomes.

## Placental Development: A Role for Inflammatory Mediators

In a healthy pregnancy, the maternal immune system adapts to protect the semi-allogeneic fetus and placenta. Circulating levels of both cytokines [e.g., interferon (IFN)-γ, tumor necrosis factor (TNF), etc.] and components of the complement system (e.g., C3a, C5a, etc.) are altered across normal pregnancy (Kraus et al., 2010; Regal et al., 2015). Several cell types in the placenta including maternal and fetal-derived placental cells, as well as specialized immune cells like decidual natural killer cells (dNK), produce, express and/or secrete inflammatory cytokines and complement regulatory proteins at the materno-fetal interface in a healthy pregnancy (Bowen et al., 2002; Weckman et al., 2018). These inflammatory mediators play a dual role in immunity and processes of normal placental development including extravillous trophoblast proliferation and invasion necessary for uterine spiral artery remodeling (Albieri et al., 1999; Bowen et al., 2002; Hanna et al., 2006; Bulla et al., 2008; Pollheimer et al., 2018). Furthermore, dNKs play an important role in placental vascular development *via* the production of angiogenic factors including VEGF, PlGF, Ang-1, and Ang-2 (Hanna et al., 2006; Le Bouteiller, 2013). Trophoblasts also increase VEGF production in response to cytokine stimulation, and monocytes will increase production of sFlt-1 in response to complement activation (Choi et al., 2002; Girardi et al., 2006; Conroy et al., 2009). Inflammatory and angiogenic systems are interdependent and tightly regulated across pregnancy. Together, their regulation is critical to placental vascular development. Therefore, disruption of either system could lead to a cascade of downstream events with negative impacts on placental vascular development and birth outcomes.

## Abnormal Placental Vascular Development Underlies Pregnancy Complications

There is abundant evidence for defective maternal spiral artery remodeling (Khong et al., 1986; Hustin et al., 1990; Pijnenborg et al., 1991; Kingdom et al., 2000; Romero et al., 2011; Fisher, 2015; Burton and Jauniaux, 2018; Pollheimer et al., 2018), abnormal villous development and vascularization (Jackson et al., 1995; Kingdom and Kaufmann, 1997; Kingdom et al., 2000; Vedmedovska et al., 2011; Burton and Jauniaux, 2018; Silver, 2018; Travaglino et al., 2019), and impaired umbilical blood flow (Trudinger et al., 1985; Salafia et al., 1997; Ferrazzi et al., 2000; Zhu et al., 2016) in a range of pregnancy outcomes with placental pathologies including preeclampsia, SGA, PTB, spontaneous abortion, and stillbirth.

Dysregulation of specific angiogenic mediators has also been associated with placental insufficiency and adverse pregnancy outcomes. The balance between angiogenic and anti-angiogenic factors, and the resulting alterations to placental vasculature, is modulated by multiple factors including oxygen homeostasis, external agents (e.g., drugs), infection, and inflammation (Khaliq et al., 1999; Kaufmann et al., 2004; Girardi et al., 2006; Conroy et al., 2013; McDonald et al., 2018; Mohammadi et al., 2018). Seminal preclinical studies established the importance of a precise angiogenic balance *in utero*, as both an absence and excess of VEGF and the angiopoietins were associated with abnormal embryonic development or lethality (Carmeliet et al., 1996; Suri et al., 1996; Maisonpierre et al., 1997; Miquerol et al., 2000). These findings have been extended to human studies, where multiple adverse pregnancy outcomes have been linked to dysregulation of circulating levels of angiogenic mediators critical for normal placental development including VEGF, PlGF, and sFlt-1, Ang-1 and -2, and soluble endoglin (Sharkey et al., 1996; Levine et al., 2004, 2006; Venkatesha et al., 2006; Chaiworapongsa et al., 2009; Romero et al., 2010; Conroy et al., 2013, 2017; Darling et al., 2014; McDonald et al., 2015b, 2018; Straughen et al., 2017).

As discussed above, there is a tight interrelationship between inflammatory pathways and angiogenic balance during placental development. Consequently, adverse pregnancy outcomes including spontaneous PTB and SGA have been associated with simultaneous dysregulation of both pathways (Girardi et al., 2006; Conroy et al., 2013; Darling et al., 2014; McDonald et al., 2015b). These data strongly support a role for disrupted placental development, due to inflammatory and angiogenic dysregulation at the materno-fetal interface, as a common pathway in the pathobiology of adverse birth outcomes.

## Maternal Infection Disrupts Placental Vascular Development

An important contributor to disruption of the inflammatory and angiogenic environment at the materno-fetal interface is maternal infection. Infections due to the TORCH pathogens [*Toxoplasma gondii*, others, rubella virus, cytomegalovirus (CMV), and herpes simplex virus] may result in adverse pregnancy outcomes *via* vertical transmission to the fetus (Coyne and Lazear, 2016). However, even in the absence of congenital infection, maternal infections such as malaria and HIV have been linked with adverse birth outcomes including PTB, LBW, SGA, and stillbirth (Desai et al., 2007; Wedi et al., 2016; Rogerson et al., 2018). Despite increased coverage of treatment for infections in pregnancy such as malaria and HIV, rates of adverse birth outcomes remain high (Madanitsa et al., 2016; Santosa et al., 2019), and a better understanding of the pathophysiology underlying infection-induced adverse birth outcomes is needed. Increasing evidence suggests placental vascular pathology may be an important contributor to the link between maternal infection and adverse birth outcomes.

A growing body of evidence has linked maternal infection with abnormal placental pathology and altered maternal and fetal hemodynamics. Histopathological examination of human placentas have reported abnormal placental villous architecture and maternal vascular malperfusion in the context of viral, bacterial and parasitic maternal infections (Carmona-Fonseca et al., 2013; Ahmed et al., 2014; Chaikitgosiyakul et al., 2014; Kim et al., 2015; Kalk et al., 2017; Ribeiro et al., 2017; Moeller et al., 2018). Preclinical studies have also demonstrated significantly altered placental vascularization, vascular remodeling, and oxygen transport in response to maternal infection (Tabata et al., 2012; Conroy et al., 2013; Hirsch et al., 2018; McDonald et al., 2018; Phillips et al., 2018). In humans, maternal infections including influenza, *Helicobacter pylori*, malaria, and HIV have been associated with impaired maternal and fetal hemodynamics (e.g., high arterial resistance) (Dorman et al., 2002; Griffin et al., 2012; Hernandez-Andrade et al., 2014; McClure et al., 2014; Di Simone et al., 2017; Ome-Kaius et al., 2017). The available evidence implicates abnormalities in placental vascularization and function as a common driver behind infection-induced adverse birth outcomes, even in non-congenital infections.

**Figure 2:**
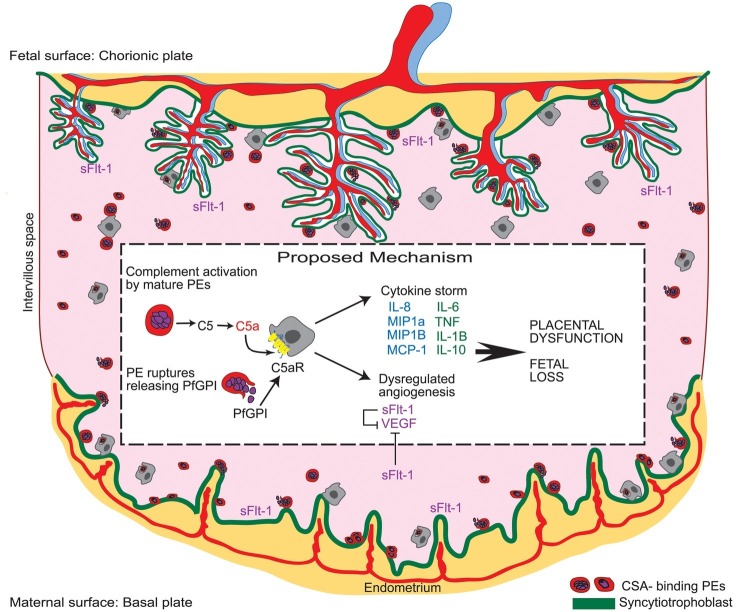
Malaria in pregnancy as a model for infection-driven dysregulation of placental vascular development as a common mechanism underlying adverse birth outcomes. During malaria in pregnancy, parasitized erythrocytes (PE) activate the maternal complement cascade. Complement component C5a acts synergistically with circulating parasite-associated bioactive molecules (i.e., *Plasmodium falciparum* glycosylphosphatidylinositol anchor molecules; *Pf*GPI) to trigger an inflammatory response at the materno-fetal interface. This inflammatory host response dysregulates the tight balance of angiogenic factors required for placental vascular development leading to aberrant placental vascularization, placental dysfunction, and adverse birth outcomes. Existing evidence for other infections during pregnancy supports this model as a common mechanism underlying adverse birth outcomes in maternal infection [reproduced from Conroy et al., 2009].

Mechanistically, dysregulation of angiogenic mediators including sFlt-1, Ang-1, and -2, soluble endoglin, and PlGF and altered placental vascular structure and function has been reported in the context of maternal infections (e.g., malaria, HIV, CMV, and acute pyelonephritis) associated with adverse birth outcomes (Chaiworapongsa et al., 2010; Silver et al., 2010, 2011; Conroy et al., 2013, 2017; Ataíde et al., 2015; Gustafsson et al., 2015; McDonald et al., 2018). Inflammatory mediators including cytokines [e.g., interleukin (IL)-1, INF-γ, and TNF] and the complement system exhibit regulatory cross-talk with angiogenic factors critical to placental development (Naldini and Carraro, 2005; Fiedler and Augustin, 2006; Girardi et al., 2006; Conroy et al., 2009, 2013). There is evidence for systemic inflammation in bacterial, viral, and parasitic maternal infections (Horton et al., 2008; Conroy et al., 2013; Cérbulo-Vázquez et al., 2014; Romero et al., 2016; Fried et al., 2017; Wilkinson et al., 2017; Harjunmaa et al., 2018; McDonald et al., 2019), and statistical modeling suggests a hierarchical relationship between dysregulated inflammation, angiogenesis, and adverse birth outcomes (Conroy et al., 2013). Collectively, these studies suggest inflammation-mediated dysregulation of the tight angiogenic balance required for placental development as a shared mechanism underlying adverse birth outcomes in maternal infection (Figure 2).

## Fetal Development is Impacted by Dysregulation of the Inflammatory-Angiogenic Axis in Maternal Infection

Many pathways critical to placental vascular development are mirrored in fetal vascular development. Normal vasculogenesis, angiogenesis, and materno-fetal hemodynamics are critical to organ growth *in utero*. Mounting evidence supports the hypothesis that disruption of these processes *via* maternal immune activation may cause defects in development of the fetal lungs, heart, and brain, with long-term consequences for offspring (Bilbo and Schwarz, 2009; McAdams et al., 2012; Burton and Jauniaux, 2018). The nature of the disruption to fetal development may depend on timing of infection. For example, studies suggest disruption to placental development and hemodynamics, especially in the first month of pregnancy, could be reflected in fetal cardiac abnormalities and congenital heart disease (Linask, 2013; Linask et al., 2014; Burton and Jauniaux, 2018). Dysregulation of inflammatory and angiogenic factors (i.e., *via* maternal infection) during a critical period of lung development is associated with bronchopulmonary dysplasia and increased neonatal mortality in the first 28 days of life (Thébaud and Abman, 2007; McAdams et al., 2012; Procianoy et al., 2015). Furthermore, a large and rapidly growing body of evidence has linked maternal immune activation and dysregulation of angiogenesis with impaired fetal neurodevelopment and long-lasting neurocognitive and neuropsychiatric sequelae for offspring (Knuesel et al., 2014; Estes and McAllister, 2016; Brown and Meyer, 2018).

Neurodevelopment is an intricate and strictly orchestrated process. Proper molecular signaling during critical gestational and postnatal periods is required for the establishment of effective neural networks (Stiles and Jernigan, 2010). Immune cells and cytokines such as microglia, complement, and IL-6 play an integral role in mediating these signals, and are tightly regulated at the materno-fetal interface (Billiards et al., 2006; Smith et al., 2007; Bilbo and Schwarz, 2009; Gallagher et al., 2013; McDonald et al., 2013; Graham et al., 2018; Prins et al., 2018; Rudolph et al., 2018; Spann et al., 2018; Rasmussen et al., 2019). In response to bacterial, viral, and parasitic infections, the maternal immune system is activated, exposing the fetus to cytokines and immune cells that are capable of passing through the immature fetal blood brain barrier and potentially impacting fetal neurodevelopment (Dammann and Leviton, 1997; Prins et al., 2018).

Evidence of the association between maternal immune activation and neuropsychiatric disorders, including schizophrenia (SZ), autism spectrum disorder (ASD) and bipolar disorder (BD), has accumulated in both epidemiological studies and preclinical models (Knuesel et al., 2014; Estes and McAllister, 2016; Brown and Meyer, 2018). Maternal immune activation *via* individual cytokines (e.g., IL-6 and IL-2), viral/bacterial mimics [e.g., polyinosinic:polycytidylic acid, poly(I:C); lipopolysaccharides, LPS], and actual infections (e.g., malaria, influenza, urinary tract infections) have been associated with long-term behavioral consequences for exposed offspring (Cai et al., 2000; Ponzio et al., 2007; Smith et al., 2007; Giovanoli et al., 2013; Knuesel et al., 2014; McDonald et al., 2015a; Choi et al., 2016; Brown and Meyer, 2018). Altered expression of proinflammatory mediators with dual roles in inflammation and neurodevelopment have been implicated in this link. Proinflammatory cytokine IL-6, which induced psychiatric behavioral outcomes in a seminal preclinical study of maternal immune activation (Smith et al., 2007), also has roles in neurogenesis, synapse formation, white matter development, and dendritic spine architecture (Wei et al., 2012; Gallagher et al., 2013; Rasmussen et al., 2019). Complement components including C5a, C3, and C1q also have well-characterized dual roles in maternal response to infection and neurodevelopment (i.e., synaptic pruning), and dysregulation of complement has been implicated in maternal infection-associated neurocognitive deficits in offspring (Stevens et al., 2007; Schafer et al., 2012; McDonald et al., 2015a; Weckman et al., 2018).

Furthermore, evidence points toward a direct connection and cross-talk between pathways critical to angiogenesis and neurodevelopment; proteins including VEGF family members have important roles in both processes (Carmeliet, 2003). Interestingly, VEGF participates in signaling required for the intricate growth and patterning of nerves and blood vessels alongside one another in the developing brain (Carmeliet, 2003). This indicates that disruption of the angiogenic environment (possibly downstream of maternal infection and inflammation) at the materno-fetal interface could also impact neurocognitive development with potential long-term neurological consequences for the offspring. In support of this contention, micro-CT images of malaria-exposed murine offspring showed alterations to fetal neurovasculature that was C5a-C5aR signaling-dependent (McDonald et al., 2015a). Since C5a activation seems to be upstream of angiogenic dysregulation in malaria in pregnancy (Figure 2; Conroy et al., 2009, 2013), these preclinical data support a model whereby maternal host response to infection in pregnancy could induce disruptions to fetal neurodevelopment *via* both angiogenic and inflammatory mechanisms.

## Abnormal Placental Development as a Driver of Infection-Induced Adverse Birth Outcomes has Implications for Treatment Strategies

An increased understanding of the impact of maternal infections on placental vascular development has implications for new intervention strategies to reduce adverse birth outcomes. For example in maternal HIV infection, despite antiretroviral treatment women exhibited dysregulation of angiogenic factors (i.e., increased circulating soluble endoglin and decreased PlGF concentrations, resulting in an anti-angiogenic state) that were associated with PTB, SGA and stillbirth (Conroy et al., 2017). Women receiving antiretrovirals and cotrimoxazole that were co-infected with HIV and malaria exhibited systemic inflammation (i.e. increased soluble TNF receptor-2) that was also associated with PTB (McDonald et al., 2019). Persistent dysregulation of inflammatory and angiogenic pathways critical to placental vascular development could explain why rates of adverse birth outcomes remain high even in the face of appropriate antimicrobial therapy and indicate that alternative strategies, including modifying host response pathways, may be necessary to reduce poor birth outcomes.

Considering the early stage at which placental vasculature is established, the timing of infection during pregnancy has important implications for prevention/treatment strategies. Increasing evidence suggests that early infection with malaria in pregnancy increases the risk of adverse birth outcomes including SGA *via* disruption to placental development (Griffin et al., 2012; Moeller et al., 2018). Since current malaria drug-based prevention strategies are not implemented until the second trimester, this could represent an important gap in the prevention of infection-induced dysregulation of placental development and resulting adverse birth outcomes (Huynh et al., 2015). The use of combined antiretroviral therapy (cART) in pregnancy has also been associated with dysregulated angiogenesis, compromised placental vascular development, and adverse birth outcomes, especially when initiated early in pregnancy (Mohammadi et al., 2018). In pre-clinical studies, supplementing cART-treated mice with progesterone prevented placental abnormalities, indicating that this addition to current treatment strategies might improve pregnancy outcomes by targeting dysregulated placental vascular development. In support of this contention, a study in a large cohort of women in Papua New Guinea demonstrated the ability of sulphadoxine-pyrimethamine and azithromycin to improve adverse birth outcomes by regulating inflammatory (e.g., C-reactive protein) and angiogenic factors (e.g., soluble endoglin, sFlt-1) critical to placental vascular development (Unger et al., 2019).

## Conclusions

Despite Millennium and Sustainable Development Goals to improve maternal-child health, the global burden of adverse birth outcomes remains high. This is in part due to a critical knowledge gap in our understanding of the mechanisms underlying adverse birth outcomes, and consequently limited or ineffective strategies. Several lines of evidence suggest that infection-driven dysregulation of inflammation and angiogenesis at the materno-fetal interface is a common mechanism underlying inadequate placental and fetal development, and adverse birth outcomes. Placental vascular development and vascular adaptation requires tight temporal and spatial regulation of cytokines, the complement system, and angiogenic factors including the VEGF and angiopoietin families. Dysregulation of those systems in the context of maternal infection leads to aberrations in spiral artery remodeling, placental vascularization and villous architecture, and deleterious materno-fetal hemodynamics that are associated with adverse birth outcomes. Current interventions for infections in pregnancy including malaria and HIV target only the pathogen and not the host response that may drive poor birth outcomes. This may explain, at least in part, why rates of adverse birth outcomes remain high. Further, dysregulation of inflammatory and angiogenic factors at the materno-fetal interface can lead to impairments in fetal neurodevelopment including neurogenesis and neurovascular development, with long term cognitive and behavioral sequelae for offspring. Considering the importance of the relationship between infection-induced dysregulation and adverse birth outcomes, future research should focus on therapeutics that target early placental development as a strategy to reduce the global burden of adverse birth outcomes.

## Author Contributions

CM and KK managed project conception and oversight. AW, MN, JW, and CM contributed to research, analysis, and writing of the manuscript. All authors contributed to manuscript revisions and approved the final manuscript.

### Conflict of Interest Statement

The authors declare that the research was conducted in the absence of any commercial or financial relationships that could be construed as a potential conflict of interest.

## Abbreviations

Ang-1 and -2: Angiopoietin-1 and -2
cART: Combined antiretroviral therapy
CMV: Cytomegalovirus
dNK: Decidual natural killer cells
IL: Interleukin
INF: Interferon
LBW: Low birth weight
NO: Nitric oxide
PlGF: Placental growth factor
PTB: Preterm birth
sFlt-1: Soluble fms-like tyrosine kinase-1
SGA: Small-for-gestational age
TNF: Tumor necrosis factor
VEGF: Vascular endothelial growth factor.
